# Cytokine treatment optimises the immunotherapeutic effects of umbilical cord-derived MSC for treatment of inflammatory liver disease

**DOI:** 10.1186/s13287-017-0590-6

**Published:** 2017-06-08

**Authors:** Samantha F. H. de Witte, Ana M. Merino, Marcella Franquesa, Tanja Strini, Johanna A. A. van Zoggel, Sander S. Korevaar, Franka Luk, Madhu Gargesha, Lisa O’Flynn, Debashish Roy, Steve J. Elliman, Philip N. Newsome, Carla C. Baan, Martin J. Hoogduijn

**Affiliations:** 1 Nephrology and Transplantation, Department of Internal Medicine, Erasmus MC, Postbus 2040, 3000 CA Rotterdam, The Netherlands; 2 Experimental Urology Department, Department of Internal Medicine, Erasmus MC, Rotterdam, The Netherlands; 3 Orbsen Therapeutics Ltd, Galway, Ireland; 4 National Institute for Health Research (NIHR) Birmingham Liver Biomedical Research Unit and Centre for Liver Research, University of Birmingham, Birmingham, UK; 5 BioInVision Inc, Mayfield Village, OH USA

**Keywords:** Immunogenic, Immunomodulatory, Mesenchymal stromal cell, Optimising

## Abstract

**Background:**

Mesenchymal stromal cells (MSC) possess immunomodulatory properties and low immunogenicity, both crucial properties for their development into an effective cellular immunotherapy. They have shown benefit in clinical trials targeting liver diseases; however the efficacy of MSC therapy will benefit from improvement of the immunomodulatory and immunogenic properties of MSC.

**Methods:**

MSC derived from human umbilical cords (ucMSC) were treated for 3 days in vitro with various inflammatory factors, interleukins, vitamins and serum deprivation. Their immunogenicity and immunomodulatory capacity were examined by gene-expression analysis, surface-marker expressions, IDO activity, PGE_2_ secretion and inhibition of T cell proliferation and IFNγ production. Furthermore, their activation of NK cell cytotoxicity was investigated via CD107a expression on NK cells. The immunomodulatory capacity, biodistribution and survival of pre-treated ucMSC were investigated in a CCl_4_-induced liver disease mouse model. In addition, capacity of pre-treated MSC to ameliorate liver inflammation was examined in an ex vivo liver inflammation co-culture model.

**Results:**

IFN-γ and a multiple cytokine cocktail (MC) consisting of IFN-γ, TGFβ and retinoic acid upregulated the expression of immunomodulatory factor PD-L1 and IDO activity. Subsequently, both treatments enhanced the capacity of ucMSC to inhibit CD4 and CD8 T cell proliferation and IFN-γ production. The susceptibility of ucMSC for NK cell lysis was decreased by IFN-β, TGFβ and MC treatment. In vivo, no immunomodulation was observed by the ucMSC. Four hours after intravenous infusion in mice with CCl_4_-induced inflammatory liver injury, the majority of ucMSC were trapped in the lungs. Rapid clearance of ucMSC(VitB_6_), ucMSC(Starv + VitB_6_) and ucMSC(MC) and altered bio-distribution of ucMSC(TGFβ) compared to untreated ucMSC was observed. In the *e*x vivo co-culture system with inflammatory liver slices ucMSC(MC) showed significantly enhanced modulatory capacity compared to untreated ucMSC.

**Conclusions:**

The present study demonstrates the responsiveness of ucMSC to in vitro optimisation treatment. The observed improvements in immunomodulatory capacity as well as immunogenicity after MC treatment may improve the efficacy of ucMSC as immunotherapy targeted towards liver inflammation.

**Electronic supplementary material:**

The online version of this article (doi:10.1186/s13287-017-0590-6) contains supplementary material, which is available to authorized users.

## Background

Mesenchymal stromal cells (MSC) are a novel therapeutic option for inflammatory conditions and have been studied in animal models as well as in preliminary clinical studies [1–6]. In particular, MSC have shown potential in clinical trials of patients with liver diseases, such as liver cirrhosis [7–10] and acute-on-chronic liver failure [11–13]. In addition, they have shown to be able to modulate the immune system in other various diseases, however, there is not convincing evidence for all diseases [1]. MSC possess two immunological properties that make them interesting candidates for cellular immunotherapy; their immunomodulatory capacity and low recognition by the host immune system. Optimisation of these properties will enhance their efficacy as immunotherapy.

MSC express a vast range of factors which target immune cells and influence their function, such as modulation of B cell and natural killer (NK) cell function, suppression of T cell proliferation/activation as well as induction of regulatory T cells (Treg) [14–17]. One of their key immunomodulatory factors is indoleamine 2,3-dioxygenase (IDO), which deprives the environment of L-tryptophan thereby inhibiting lymphocyte proliferation [18], has been demonstrated to promote graft acceptance after solid organ transplantation [19]. In addition, prostaglandin E_2_ (PGE_2_), CCL2 and interleukin 1 receptor antagonist (IL1RA), which are produced by MSC have shown to have an important role in the therapeutic effects of MSC on autoimmune diseases, such as rheumatoid arthritis [3, 4, 20–23]. Besides secretion of anti-inflammatory factors, MSC express cell surface proteins that are known for their immunoregulatory function, such as programmed death-ligand 1 (PD-L1), which inhibits lymphocyte proliferation [24].

In addition, MSC have low immunogenicity due to low levels of HLA class I and lack of HLA class II and co-stimulatory molecule expression on their surface, which may potentially allow the use of allogeneic MSC for treatment without inducing allogeneic responses [25]. In addition, complex formation of HLA class I with inhibitory NK cell receptors protects MSC from NK cell lysis [26]. However, HLA class I enables recognition of MSC by allogenic CD8^+^ T cells, and initiation of lysis [26, 27] and Cho et al. observed IgG antibody responses to allogeneic umbilical cord tissue-derived stromal cells [28]. Consequently, recognition by immune cells in vivo may reduce the survival time of MSC and thereby restricting the duration of their effect [29]. Another in vivo drawback is that MSC get trapped in the lungs due to their size, restricting their migration to the site of injury [29, 30]. Improving MSC ex vivo in such a way that their immunogenicity is reduced and their immunosuppressive capacity is enhanced may therefore be beneficial for their survival as well as their effectiveness after administration.

MSC can be isolated from different tissues in the body [31], and often constitute a heterogeneous population of cells. The use of a more homogeneous MSC subset, will potentially increase the consistency of treatment results and improve efficacy. Studies involving specific MSC subsets, based on selection of MSC expressing Stro-1, CD73, CD90 or CD271, have shown that these subsets possess enhanced immunosuppressive capacities [32–36]. Recently, a new subset, identified by the surface marker CD362^+^ (Syndecan-2) has been identified (patent number WO 2013117761 A1), which showed improved clonogenicity and immunomodulatory properties.

MSC contain an abundance of surface receptors for a large variety of factors such as inflammatory factors, interleukins, prostaglandins and several vitamins. Treatment of MSC by such factors, like interferon gamma (IFN-γ), tumour necrosis factor alpha (TNF-α) and interleukin 17 (IL-17), induces MSC to adapt towards a more immunomodulatory phenotype and alters their immunogenicity [37–39]. In addition, different culture conditions, such as serum-deprivation and hypoxia [40] may affect the function of MSC.

In this study, we exposed MSC to various growth factors, cytokines and culture conditions with the aim to generate MSC with enhanced properties; reduced immunogenicity and improved immunosuppressive capacity, for immunotherapy to target liver inflammation. Through analysing surface marker expression, gene expression and secretome we examined the immunophenotype of pre-treated MSC. In addition, the immunogenicity and immunomodulatory capacity of pre-treated MSC were evaluated by their susceptibility to trigger NK cytotoxic activity and their capacity to inhibit lymphocyte proliferation, respectively. Furthermore, their potential to ameliorate inflammation and immunomodulate the immune response was tested in vivo as well as ex vivo in a murine liver inflammation model.

## Methods

### Culture expansion of ucMSC

Human umbilical cord tissue was collected from Caesarean section deliveries by Tissue Solutions Ltd. (Glasgow, UK) from virally screened healthy donors. All such cord was obtained according to legal and ethical requirements of the UK, with the approval of the relevant ethics committee and with anonymous consent from the donor. Cords were harvested within 48 hours of birth and the average length of collected umbilical cord tissue (n = 4–6) was 8.6 cm, with all tissues transported for processing to Orbsen Therapeutics Ltd. in AQIX® solution (London, UK) at 4 °C. In short, umbilical cord tissue was washed and the whole tissue was manually dissociated before enzymatic digestion in an enzyme cocktail consisting of MEM alpha (Gibco, Thermo Fisher Scientific, Paisley, UK), collagenase 1 (2 mg/ml), hyaluronidase 1 (1 mg/ml) and DNase (0.1 mg/ml) (Sigma-Aldrich, Arklow, Ireland) for a maximum of 2 hours at 37 °C. Thereafter, a single cell suspension was obtained by filtration (100 μm) and the cells were stained with CD362 (Syndecan-2/ORB, APC, clone 305515, dilution 1:50, R&D Systems, Minneapolis, MN, USA) for 30 minutes at 4 °C. Subsequently, cells were washed and resuspended in MACs buffer 80 μl/10^7^cells. Next, the cells were incubated with anti-APC beads (20 μl/10^7^cells, Miltenyi Biotec, GmbH, Bergisch Gladbach, Germany) for 15 minutes at 4 °C. CD362^+^ cells were then isolated using MS MACs column according to manufacturer’s instructions, (Miltenyi Biotec). Each cell fraction was counted, seeded for expansion and cryopreserved at passage 2 for shipment to Erasmus Medical Center. Here ucMSC were cultured in minimum essential medium Eagle alpha modification (MEM-α; Sigma-Aldrich, St. Louis, MO, USA) containing 2 mM L-glutamine (Lonza, Verviers, Belgium), 1% penicillin/streptomycin solution (P/S; 100 IU/ml penicillin, 100 IU/ml streptomycin; Lonza) and supplemented with 15% fetal bovine serum (FBS; Lonza) and 1 ng/ml basic fibroblast growth factor (bFGF) (Sigma-Aldrich) and kept at 37 °C, 5% CO_2_ and 20% O_2_. Medium was changed once a week and ucMSC were passaged at approximately 80–90% confluence using 0.05% trypsin-EDTA (Life Technologies, Paisley, UK). All ucMSC used in experiments were between passage 4 and 7.

### Study design

A set of in vitro, in vivo and ex vivo experiments was designed to analyse the effect of pre-treatment of ucMSC with various growth factors, cytokines and culture conditions, as depicted in Additional file 1: Figure S1 and described in detail below.

### Treatment of ucMSC

UcMSC were treated with (Table 1 and Additional file 2: Table S1): activin A (12 ng/ml; Miltenyi Biotec GmbH), budesonide (50 mM; FLUKA Sigma-Aldrich), interferon gamma (IFN-γ, 50 ng/ml; Life Technologies), interferon beta (IFN-β, 5 ng/ml; Peprotech, Rocky Hill, NJ, USA), transforming growth factor beta 1 (TGFβ, 10 ng/ml; R&D Systems), interleukin-15 (IL-15, 10 ng/ml; Peprotech), interleukin-17 (IL-17, 50 ng/ml; Peprotech), retinoic acid (RA, 1 μM and 10 μM; Sigma), trespostinil (20 ng/ml; Cayman Chemical Company, Ann Arbor, MI, USA), tumour necrosis factor alpha (TNF-α, 10 ng/ml; Peprotech), 1α,25-dihydroxyvitamin D_3_ (vitamin D_3_, VitD_3_; 0.01 μM; Sigma-Aldrich) and pyridoxal hydrochloride (vitamin B_6_, VitB_6_, 1 μM; Sigma-Aldrich). All factors were added at day 0 and the cells were analysed at day 3, except for trespostinil, which due to its instability was added daily. In addition, ucMSC were also cultured without serum (starvation, Starv). Two further combinations were also used: starvation and vitamin B_6_ (Starv + VitB_6_) and the multiple cytokine combination (MC), consisting out of IFN-γ, TGFβ and RA.

**Table 1:** Observed changes in immunogenic and immunomodulatory molecules after 3 days in vitro treatment of ucMSC

	Immunogenic read out parameters		Immunomodulatory read out parameters				Fold increase of gene expression compared to unstimulated
	HLA class I- expressing cells [%]	HLA class II- expressing cells [%]	L-Kynurenine (correlated IDO activity) [μM]	PGE_2_ [ng/ml]	PD-L1- expressing cells [%]	CD73-expressing cells [%]	IL-1RA [corrected for GAPDH]
[-]	7 ± 1	10 ± 3	2 ± 5	14 ± 3	46 ± 6	95 ± 2	1 ± 0
IFN-y	60 ± 9^*^	68 ± 9^*^	27 ± 10^*^	1 ± 0^*^	95 ± 3^*^	94 ± 3	1 ± 0
IFN-β	36 ± 12^*^	14 ± 5	10 ± 10	3 ± 2	73 ± 12	91 ± 5	4 ± 1
TGFβ	9 ± 3	10 ± 3	3 ± 1	3 ± 2	58 ± 7	89 ± 5	4 ± 4
Starv	15 ± 3^*^	4 ± 2	6 ± 3	4 ± 3	24 ± 8^*^	100 ± 0	1 ± 0
VitB_6_	47 ± 5^*^	34 ± 9^*^	3 ± 5	12 ± 5	59 ± 14	100 ± 0	67 ± 42
Starv + VitB_6_	62 ± 7^*^	22 ± 5^*^	0 ± 3	6 ± 3	72 ± 7^*^	100 ± 0	507 ± 262
RA	26 ± 5^*^	18 ± 5	0 ± 0	5 ± 2	59 ± 11	98 ± 1	5 ± 4
MC	85 ± 4^*^	76 ± 7^*^	29 ± 8^*^	8 ± 3	97 ± 1^*^	98 ± 2	3 ± 2

The table displays the mean ± SEM percentages of MSC-expressing surface markers HLA class I and II, PD-L1 and CD73, measured by flow cytometry , the mean ± SEM concentration of L-Kynurenine [μM] and PGE_2_ [ng/ml] measured by ELISA and fold increases in IL1RA mRNA expression compared to unstimulated MSC. *n* = 5

*ucMSC* umbilical cord-derived mesenchymal stromal cells, *HLA* human leukocyte antigen, *IDO* indoleamine 2,3 dioxygenase, *PGE*_*2*_ prostaglandin E_2_, *PD-L1* programmed cell death ligand 1, *IL* interleukin, *IFN-γ* interferon gamma, *IFN-β* interferon beta, *TGFβ* transforming growth factor beta, *Starv* starvation, *VitB*_*6*_ Vitamin B_6_, pyridoxal hydrochloride, *RA* retinoic acid, *MC* multiple cytokine combination

^*^Indicates *p* < 0.05

### Flow cytometric analysis

Flow cytometric characterisation of control and treated ucMSC was performed by labelling with standard MSC markers, according to ISSCT criteria [41]: CD13 (PE-Cy7, BD Biosciences, San Jose, CA, USA), CD73 (PE, BD Pharmingen, San Diego, CA, USA), CD90 (APC, R&D Systems) and CD105 (FITC, R&D Systems), hematopoietic markers CD31 (PB, BD Biosciences), CD45 (APC-Cy, BD Biosciences) and the surface markers: HLA class I (Pacific Blue, Biolegend, San Diego, CA, USA), HLA class II (PERCP, BD Biosciences), CD362 (APC, R&D Systems), PD-L1 (PE, Biolegend), CD271 (PE-Cy7, BD Pharmingen), CD73 (PE, BD Pharmingen) and CD40 (PERCP, Biolegend).

### L-Kynurenine assay

IDO activity was analysed by measuring L-Kynurenine levels in the supernatants. Supernatant was harvested at day 3 and centrifuged for 10 minutes at 3000 rpm, to remove dead cells. Samples were stored at −80 °C until the assay was performed. After thawing, samples were spun down, at 3000 rpm for 10 minutes. Subsequently, 100 μL 30% trichloroacetic acid (TCAA) was added to 200 μL sample, this was then vortexed and placed in a 50 °C waterbath for 30 minutes. The tubes were then centrifuged at 12,000 rpm for 5 minutes. A total of 75 μL supernatant was added to 75 μL Ehrlich reagent, in duplicate, and measured with a spectrophotometer (Victor 1420 Multilabel Plate Reader; LKB-Wallac, Turku, Finland) at an absorbance of 490 nm.

### PGE_2_ ELISA

PGE_2_ secretion by ucMSC was analysed in ucMSC culture supernatant with the PGE_2_ High-sensitivity ELISA Kit according to the manufacturer’s instructions (Enzo Life Sciences BVBA, Antwerp, Belgium, ADI-930-001).

### RT-PCR

UcMSC were seeded at 100,000 cells/well in 6-well plates and stimulated with the various factors. At day 3, supernatant was removed and cells were washed with phosphate-buffered saline (PBS), RNA (Life Technologies) was added later and the plates placed in the fridge. The next day, mRNA was isolated with the use of the High Pure RNA Isolation Kit (Roche Diagnostics, Basel, Switzerland). mRNA was isolated from liver slices using Trizol reagent (Invitrogen, Life Technologies, Carlsbad, CA, USA). cDNA was synthesized from 500 ng mRNA with random primers (Promega Benelux B.V., The Netherlands). Quantitative gene expression was determined using TaqMan Gene Expression Master Mix (Life Technologies) and assays-on-demand for IL-1RA (Hs00893626_m1), MCP-1 (Mm00441242_m1), IL-6 (Mm00446190_m1), TNF-α (Mm00443258_m1) and IP-10 (Mm00445235_m1). GAPDH (Hs 99999905_m1) was used as housekeeping gene for analysing gene expression of ucMSC. Data was expressed as the gene expression relative to GAPDH and comparing fold changes to the untreated MSC. Gene expression levels of the liver slices are expressed as copy numbers (efficiency^−∆Ct^).

### Isolation of PBMC

Peripheral blood samples were collected from living kidney donors at Erasmus MC or isolated from buffy coats obtained from Sanquin Blood Bank (Amsterdam, Netherlands). Peripheral blood mononuclear cells (PBMC) were isolated by density gradient centrifugation, using Ficoll-Paque (GE Healthcare, Chicago, IL, USA) and frozen at −180 °C until use.

### CD107a NK cytotoxicity assay

For generation of activated NK cells, PBMCs were thawed, washed and seeded at 100,000 cells/well in 96-well plates. They were cultured in RPMI 1640 medium (Life Technologies) with 1% penicillin/streptomycin solution (P/S; 100 IU/ml penicillin, 100 IU/ml streptomycin; Lonza), 10% heat-inactivated FBS (Lonza), interleukin-2 (IL-2, 2.10^2^ IU/ml; Peprotech) and interleukin-15 (IL-15, 10 ng/ml; Peprotech). At day 7, cells were collected and washed and NK cells were isolated using a negative selection MACS procedure (NK Cell Isolation Kit, human; MACS Miltenyi Biotec). Following MACS and a washing step, the cells were left overnight at 37 °C in a polystyrene tube with IL-2 and IL-15.

MACS-sorted NK cells were added to pre-treated ucMSC at a 4:1 ratio for 1 hour at 37 °C, together with monensin (0.1 μL/200 μL/well; BD Golgistop; BD Biosciences) and αCD107a antibody (LAMP-1, APC, BD Pharmingen) in polypropylene tubes. The cells were then washed and stained for CD3 (PERCP; BD Biosciences), CD16 (PE; BD Biosciences) and CD56 (PE; BD Biosciences) for 30 minutes at 4 °C. The cells were subsequently washed and analysed with the FACSCanto II flow cytometer.

### T cell proliferation assay

Pre-treated ucMSC were seeded into 96-well plates and left overnight to adhere in the incubator. The next day, PBMC were labelled with Cell Trace CFSE (Life Technologies) according to the instructions of the manufacturer and seeded on top of the ucMSC, at different [MSC:PBMC] ratios: [1:10], [1:5] and [1:2.5]. αCD3/CD28 stimulation was added (0.5 ug/ml αCD3 antibody, 0.5 μg/ml αCD28 antibody and 0.5 μg/ml goat-α-mouse antibody; Life Technologies). The co-cultures were left for 3 days and PBMC were collected. PBMC were stained for CD4 (APC; eBioscience, San Diego, CA, USA), CD8 (Pe-cy7; eBioscience) and intracellular IFN-γ (Bv421; BD Biosciences). With the use of the FACSCanto II flow cytometer the proliferation of PBMCs was measured.

#### Carbon tetrachloride (CCl_4_) liver injury model

Healthy 8-week-old male C57BL/6 mice were purchased from Charles River (Sulzfeld, Germany). The mice were housed in a facility with a 12-hour light-dark cycle and allowed free access to food and water. All the procedures and animal housing conditions were carried out in strict accordance with current EU legislation on animal experimentation and were approved by the Institutional Committee for Animal Research (DEC protocol EMC No. 127-12-14).

In the first set of experiments mice received either intraperitoneal (IP) CCl_4_ solution (4 μl/g bodyweight, 250 μl/ml mineral oil; 289116 Sigma-Aldrich) or mineral oil for controls. Three hours after CCl_4_ injection pre-treated ucMSC, which were labelled with Qtracker beads, were infused intravenously (IV) in the tail vein in 200 μl suspension in PBS (250,000 cells/mice). Four hours after ucMSC infusion, mice were sacrificed using cardiac puncture under isoflurane anaesthesia (Additional file 3: Figure S2 for experimental regimen). Whole mice were embedded in mounting medium for cryotomy (O.C.T. compound; VWR Chemicals, Radnor, PA, USA) and put in liquid nitrogen until frozen. The whole mice were then stored at -80 °C until shipment to BioInVision (Cleveland, OH, USA) for imaging.

In the second set of experiments, mice receiving IP CCl_4_ solution had a similar infusion of pre-treated ucMSC 3 hours later. After 72 hours the mice were sacrificed by cervical dislocation and blood and livers were harvested and frozen until further use.

### ALT measurement

Serum samples were thawed and centrifuged at 3000 rpm for 10 minutes. Thereafter, 50 μl of sample was diluted 1:5 with MilliQ. ALT quantification was done with an ALT measurement kit according to the instructions of the manufacturer (Roche Diagnostics; 04467388190). The samples were measured using a Roche/Hitachi cobas c analyzer.

### Labelling, quantification and visualization of MSC

Pre-treated ucMSC were collected using 0.05% trypsin-EDTA (Life Technologies) and washed. Subsequently they were labelled with Qtracker 605 cell labeling kit beads according to the manufacturer’s instructions (Life Technologies). UcMSC take up Qtracker 605 beads during the labelling procedure. Post labelling, ucMSC were washed to remove any beads that were not internalized by the ucMSC.

Three-dimensional anatomical and molecular fluorescence videos were generated of the frozen whole mice samples by BioInVision Inc. with CryoViz™ technology. The CryoViz™ technology picks up the signal of clusters of Qtracker 605 beads, which are internalized in the ucMSC. Cell counts were quantified using imaging algorithms.

### Ex vivo culture of liver slices

Mouse livers were obtained from healthy C57Bl/6 mice. Directly after isolation, mouse livers were sliced into 150 μm thick slices with a diameter of 1 cm with Vibratome (VT 1200S, Leica Biosystems, Wetzlar, Germany). The slices were subsequently placed in 6-well plates containing differently pre-treated MSC in high-glucose DMEM with 10% serum, Additional file 4: Figure S3. In addition, lipopolysaccharide [(LPS), 0.2 μg/ml; Sigma-Aldrich] was added to the medium. The tissues were kept overnight at 37 °C, 5% CO_2_ and 20% O_2_ on a shaker (50 rpm). After 24 hours, supernatant was collected and the liver slices were snap frozen.

### Milliplex assay

Serum and tissue culture supernatant levels of IL-6, IL-10, granulocyte colony-stimulating factor (G-CSF), interferon gamma-induced protein 10 (IP-10), keratinocyte chemoattractant chemokine (C-X-C motif) ligand 1 (KC, CXCL1), monocyte chemoattractant protein-1 (MCP-1, also known as CCL2), macrophage inflammatory protein-1 alpha (MIP-1α) and tumour necrosis factor alpha (TNF-α) were measured using a Milliplex mouse cytokine/chemokine magnetic bead panel multiplex assay (Merck Millipore, Billerica, MA, USA) and Mouse Premixed Multi-Analyte kit (Magnetic Luminex assay; cat LXSAMSM; R&D Systems). The samples were measured on the Luminex 100/200 cytometer (Luminex, Austin, TX, USA) using Xponent software.

### Statistical analysis

Data were analysed using IBM SPSS Statistics 21 (IBM Corp., Armonk, NY, USA) and Prism software v5.04 (GraphPad Software Inc., La Jolla, CA, USA), herein Mann-Whitney tests were performed. *P* values <0.05 were considered significant.

## Results

### Characterization of ucMSC

Flow cytometric analysis of ucMSC demonstrated expression of CD13, CD73, CD90 and CD105 and absence of CD31 and CD45 (Additional file 5: Figure S4). This is in line with the minimal ISSCT criteria and thus confirming the MSC phenotype of the used cells.

### Impact of pre-treatment of ucMSC on immunogenicity and immunomodulatory phenotype

To modulate the immunomodulatory and immunogenic phenotype of ucMSC in vitro, ucMSC were differentially treated for 3 days and the expression of key immunomodulatory and immunogenicity molecules was examined (Table 1).

Expression of HLA class I and II on the ucMSC cell surface was used as a marker of ucMSC immunogenicity. The percentage of HLA class I-expressing ucMSC increased after treatment with IFN-γ (8-fold; *p* < 0.05), IFN-β (5-fold; *p* < 0.05), Starv (2-fold; *p* < 0.05), VitB_6_ (7-fold; *p* < 0.05), Starv + VitB_6_ (9-fold; *p* < 0.05), RA (4-fold; *p* < 0.05) and MC (12-fold; *p* < 0.05) compared to untreated ucMSC. In addition, treatment with IFN-γ, VitB_6_, Starv + VitB_6_ and MC also increased the number of ucMSC expressing HLA class II by 7- (*p* < 0.05), 3- (*p* < 0.05), 2- (*p* < 0.05) and 8-fold (*p* < 0.05), respectively.

The number of ucMSC expressing the negative co-stimulatory molecule PD-L1 increased with IFN-γ (96% ± 3; *p* < 0.05), Starv + VitB_6_ (72% ± 1; *p* < 0.05) and MC treatment (97% ± 1; *p* < 0.05), compared to untreated ucMSC (46% ± 6). In addition, expression levels of PD-L1 per ucMSC were increased by IFN-γ (median fluorescence intensity (MFI) = 1947 ± 263; *p* < 0.05), Starv + VitB_6_ (MFI = 755 ± 235; *p* < 0.05) and MC treatment (MFI = 1279 ± 216; *p* < 0.05), with respect to untreated ucMSC (MFI = 219 ± 22) (data not shown in Table 1). All treatments preserved the expression of CD73, which is involved in anti-inflammatory adenosine production. Gene expression of IL1RA showed trends of upregulation after VitB_6_ treatment and the combined treatment of Starv + VitB_6_ (67- (*p* < 0.05), and 507-fold increase (*p* < 0.05), respectively). IFN-γ and MC treatment significantly upregulated IDO activity of ucMSC 14-fold (*p* < 0.05) and 15-fold (*p* < 0.05), respectively. IFN-γ treatment significantly reduced the secretion of anti-inflammatory PGE_2_ compared to untreated MSC (14-fold; *p* < 0.05).

Treatment with IL-7, IL-15, IL-17, budesonide, trespostinil, activin A and TNF-α showed no effect on any of these parameters (Additional file 2: Table S1) and thus the effects of these factors were not examined further.

### IFN-β, TGFβ and MC treatment of ucMSC protects against NK cell lysis

**Fig. 1:**
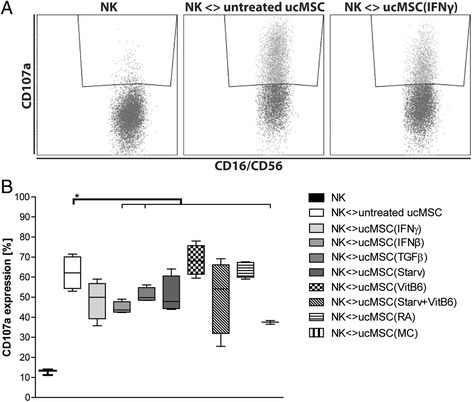
CD107a-expressing NK cells after exposure to pre-treated ucMSC. **a** Representative FACS plots of CD107a expression on NK cells, without ucMSC (*left*), with untreated ucMSC (*middle*), with IFN-γ-treated ucMSC (*right*) **b** Graph displaying boxplots of CD107a-expressing NK cells after exposure to the various pre-treated MSC (untreated, IFN-γ, IFN-β, TGFβ, starvation, vitamin B_6_, Starv + VitB_6_, retinoic acid and combination IFN-γ + TGFβ + RA (MC)). Results are shown as means ± SEM (n = 4). ^*^Indicates significant difference (*p* < 0.05). *IFN-γ* interferon gamma, *MC* multiple cytokine cocktail, *NK* natural killer cells, *RA* retinoic acid, *Starv* starvation, serum deprivation, *TGFβ* transforming growth factor beta, *ucMSC* umbilical cord-derived mesenchymal stromal cells, *VitB*_*6*_ vitamin B_6_, pyridoxal hydrochloride

To investigate the effect of pre-treatment of ucMSC on their susceptibility to NK cell lysis, the induction of CD107a expression on NK cells by ucMSC was analysed. The percentage of CD107a-expressing NK cells increased from 13% ± 1 to 62% ± 4 (*p* < 0.05) when NK cells were exposed to untreated ucMSC (Fig. 1), however pre-treatment of ucMSC with IFN-β, TGFβ and MC reduced the increase in CD107a expression on NK cells to 45% ± 1 (*p* < 0.05), 51% ± 2 (*p* < 0.05) and 37% ± 1 (*p* < 0.05) respectively.

#### Pre-treated MSC inhibit T cell proliferation and IFN-γ production

**Fig. 2:**
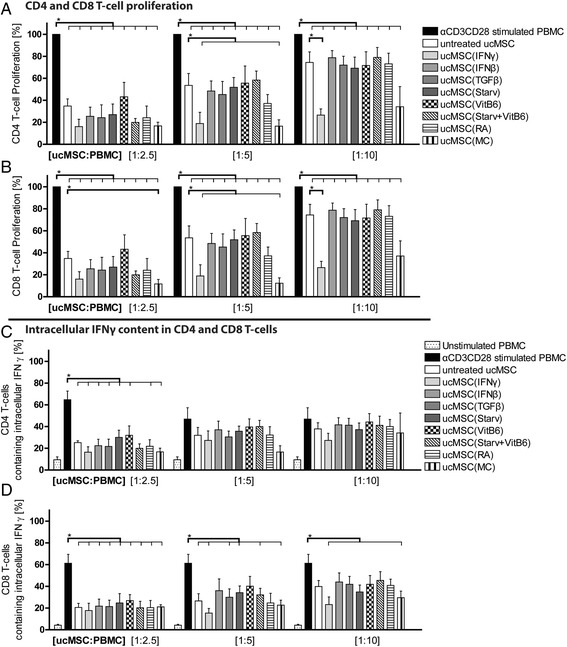
Inhibition of CD4 and CD8 T cell proliferation and intracellular IFN-γ production. MSC and αCD3CD28-stimulated and CSFE-labelled PBMCs were co-cultured at different ratios for 7 days. Thereafter proliferation of **a** CD4 and **b** CD8 T cells was measured utilizing CFSE dilution with FACS. Proliferation is expressed as the percentage of proliferating cells relative to the positive control in the absence of ucMSC. **c** IFN-γ production by CD4 and **d** CD8 T cells was measured using FACS and an intracellular labelling of IFN-γ. The IFN-γ-containing CD4 and CD8 T cells are represented as a percentage from the CD4 or CD8 T cell population. Results are shown as means ± SEM (n = 5). ^*^Indicates significant difference (*p* < 0.05). *IFN-γ* interferon gamma, *MC* multiple cytokine cocktail, *PBMC* peripheral blood mononuclear cells, *RA* retinoic acid, *Starv* starvation, serum deprivation, *TGFβ* transforming growth factor beta, *ucMSC* umbilical cord-derived mesenchymal stromal cells, *VitB*_*6*_ vitamin B_6_, pyridoxal hydrochloride

To examine the potential of MSC to inhibit CD4 and CD8 T cell proliferation and IFN-γ production, αCD3CD28-activated PBMCs were co-cultured at different ratios with pre-treated ucMSC. CD4 and CD8 T cell proliferation was inhibited in a dose-dependent manner by all ucMSC (Fig. 2a/b). UcMSC treated with IFN-γ and MC suppressed CD4 and CD8 T cell proliferation more potently than untreated MSC (Fig. 2a/b). However, none of the pre-treated ucMSC was able to significantly reduce the intracellular IFN-γ content of CD4 and CD8 T cells, compared to the untreated MSC (Fig. 2c/d).

### Effect of ucMSC in CCl_4_-induced liver inflammation model

**Fig. 3:**
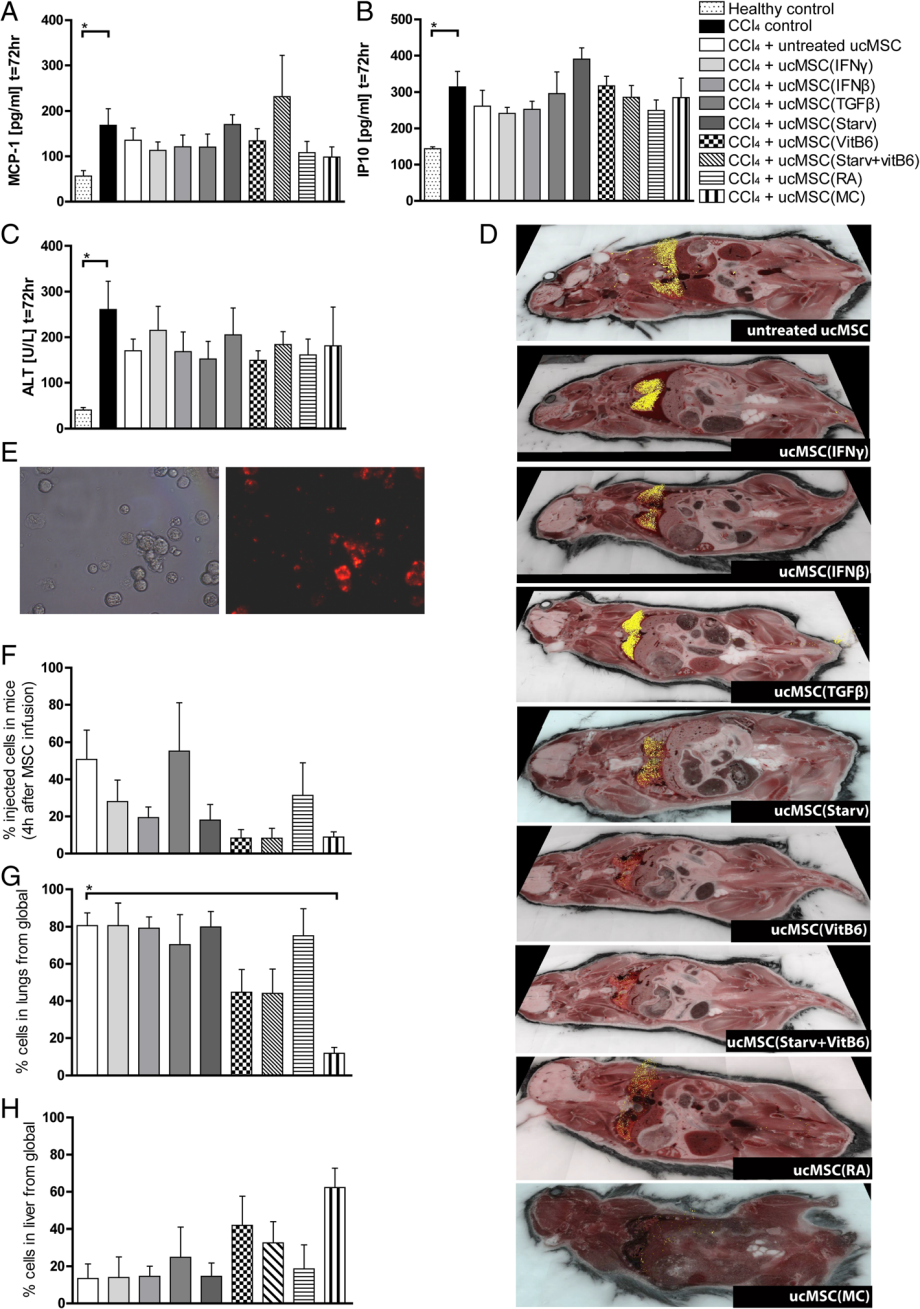
Effects on inflammation and liver damage in CCL_4_-induced liver injury and biodistribution of pre-treated MSC. Pretreated MSC were labelled with fluorescent Qtracker 605 beads and intravenously infused in C57BL/6 mice. **a** Levels of MCP-1, **b** IP-10 and **c** ALT were measured in the serum 72 hours after ucMSC infusion. **d** Representative images of the biodistribution of pre-treated MSC in vivo 4 hours after ucMSC infusion. *Yellow dots* correspond with a cluster of observed MSC. **e** Representative image of ucMSC labelled with Qtracker 605 beads, still visible in MSC 4 hours after labelling. **f** Diagram showing the percentage of infused ucMSC 4 hours after administration. Figures (**g**) and (**h**) show the relative number of ucMSC in lungs and liver, respectively, calculated from the total number of detected ucMSC. Results are shown as means ± SEM (n = 4). ^*^Indicates significant difference (*p* < 0.05) with respect to the CCL_4_ control. *ALT* alanine aminotransferase, *CCl*_*4*_ carbon tetrachloride, *IFN-γ* interferon gamma, *MC* multiple cytokine cocktail, *MCP-1* monocyte chemotactic protein-1, *RA* retinoic acid, *Starv* starvation, serum deprivation, *TGFβ* transforming growth factor beta, *ucMSC* umbilical cord-derived mesenchymal stromal cells, *VitB*_*6*_ vitamin B_6_, pyridoxal hydrochloride

To examine the immunomodulatory capacity of pre-treated ucMSC in CCl_4_-treated mice, serum levels of inflammatory associated cytokines MCP-1 and IP-10 were measured 3 days after cell infusion (Fig. 3a/b). Whilst significantly elevated levels were observed when comparing healthy to liver-injured mice, no significant differences were observed with ucMSC infusion. Similarly, infusion of pre-treated ucMSC did not reduce ALT levels as a marker of liver damage with respect to the CCL_4_ control or compared to infusion with untreated ucMSC (Fig. 3c). As no immunomodulation was observed; biodistribution of ucMSC was subsequently examined.

### Pre-treatment of ucMSC alters their retention and biodistribution in vivo

UcMSC were labelled with Qtracker605 beads to enable analysis of their biodistribution at a single cell level by CryoViz imaging. Representative bright field and fluorescent images of ucMSC, 4 hours post labelling with Qtracker605 beads are shown in Fig. 3e. Four hours after infusion of 250,000 labelled ucMSC in CCl_4_-treated mice, 51% of untreated ucMSC were detectable within the mice (Fig. 3d/f). The majority of these ucMSC were located within the lungs (81%) with a small percentage located within the liver (13%) (Fig. 3g/h). Pre-treatment with MC (12%) led to a significantly lower distribution of ucMSC to the lungs compared to untreated ucMSC (81%) (Fig. 3g). Furthermore, a larger percentage of TGFβ-treated MSC were found within mice (62%) compared to untreated ucMSC, of which a larger percentage were found in the liver (25%) compared to untreated ucMSC (13%) (Fig. 3h). These observations were not a consequence of cell size differences as MC- and TGFβ-treated ucMSC were the same size as untreated ucMSC (Additional file 6: Figure S5). Pre-treatment with IFN-γ, IFN-β, Starvation, VitB_6_ and MC resulted in the detection of lower numbers of ucMSC 4 hours after administration compared to untreated ucMSC (Fig. 3f).

### UcMSC(MC) resolve liver inflammation in an ex vivo liver tissue model

**Fig. 4:**
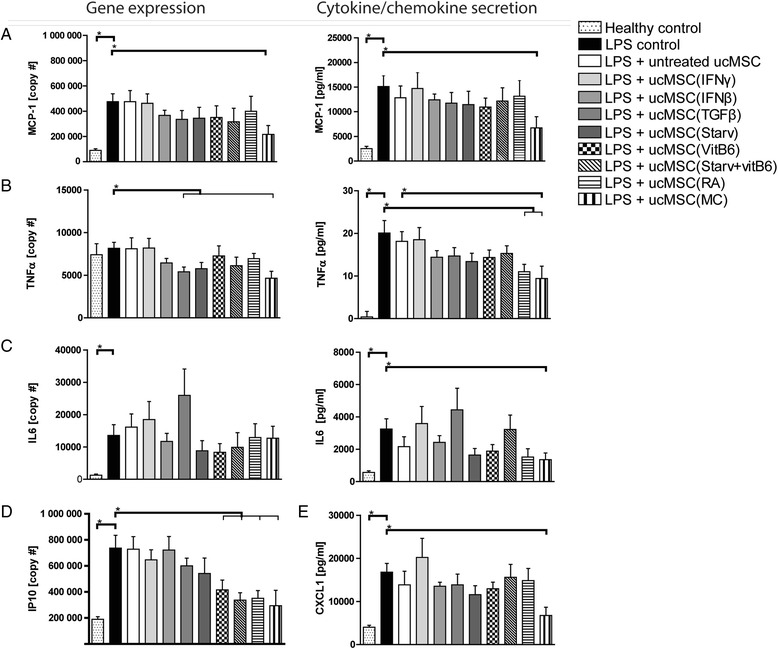
Effect of ucMSC on ex vivo liver slices. Gene expression levels and cytokine/chemokine levels were measured in liver slices treated with LPS and supernatant, respectively. (**a**)(**b**)(**c**) Both gene expression levels and secretion levels were measured for MCP-1, TNF-α and IL-6. In addition, **d** gene expression levels of IP-10 and also **e** CXCL1 were measured in the medium. Results are shown as means ± SEM (n = 6). ^*^Indicates significant difference (*p* < 0.05). *CXCL1* chemokine (C-X-C motif) ligand 1, *IFN-γ* interferon gamma, *IL* interleukin, *IP-10* interferon gamma-induced protein 10, *LPS* lipopolysaccharide, *MC* multiple cytokine cocktail, *NK* natural killer cells, *RA* retinoic acid, *Starv* starvation, serum deprivation, *TGFβ* transforming growth factor beta, *TNF-α* tumour necrosis factor alpha, *ucMSC* umbilical cord-derived mesenchymal stromal cells, *VitB*_*6*_ vitamin B_6_, pyridoxal hydrochloride

As intravenously administered ucMSC poorly distribute to the liver, we assessed the efficacy of ucMSC in ameliorating liver inflammation in an ex vivo liver tissue slice, where cells were placed in direct contact with injured liver tissue. Significantly elevated gene expression levels and secretion levels of inflammatory factors (MCP-1, TNF-α, IL-6, IP-10 and CXCL1) were observed in the diseased control liver tissue compared to the healthy control (Fig. 4). Pre-treatment of MSC with MC resulted in a marked reduction in expression levels of several inflammatory genes (MCP-1, TNF-α and IP-10), with more limited effects seen on TNF-α ( ucMSC(IFN-β)) and IP-10 (ucMSC(VitB_6_), ucMSC(starvation) and ucMSC(RA)). Moreover, MC pre-treatment decreased secretion of MCP-1, TNF-α, IL-6 and CXCL1 (all *p* < 0.05), whereas RA pre-treatment only reduced TNF-α secretion.

## Discussion

MSC have shown to be a promising cell type for treating various immune disorders in numerous disease models [3, 4, 19, 21, 22], including clinical trials of patients with liver disease [7–13]. However, the immunosuppressive effects of MSC need to be induced by inflammatory stimulation, and MSC get trapped in the lungs after intravenous infusion and have a short in vivo survival time [29, 30]. Therefore, to enhance their therapeutic efficacy, improvement of their immunomodulatory properties and immunogenicity is necessary. The present study demonstrates that ucMSC are responsive to a variety of treatments, including inflammatory factors, vitamins and serum-deprivation. Their responsiveness may be employed to improve the properties of ucMSC to generate cells with enhanced therapeutic efficacy.

In vitro experiments showed that treatment with IFN-γ or MC, increases the immunosuppressive capacity of ucMSC. In addition, treatment with IFN-β, TGFβ and MC strongly decreases their susceptibility to lysis by NK cells. In vivo tracking results showed differential effects of the pre-treatments on the MSC’s biodistribution and survival, where treatment with VitB_6_, Starv + VitB_6_ and MC ensured rapid clearance while treatment with TGFβ resulted in prolonged presence of ucMSC. No immunomodulatory effects were observed by pre-treated ucMSC in a CCl_4_ liver inflammation model, possibly due to the fact that the majority of the cells were trapped in the lungs. In the ex vivo culture system in which liver slices were cultured in the presence of ucMSC, significant amelioration of the inflammation by ucMSC(MC) was demonstrated by reduced inflammatory cytokine production. In summary, most effectively, pre-treatment of ucMSC with the multiple cytokine combination MC, resulted in enhanced immunomodulation and reduced immunogenicity.

Application of optimised ucMSC as an immunotherapy depends on the therapeutic requirements necessary to target the disorder. For example, strong immunomodulatory properties in MSC such as high IDO activity are favourable when helping graft acceptance after transplantation [19]. Secretion of PGE_2_ and CCL2 has shown to be important to attenuate sepsis, local inflammation and mediates autoimmune disorders [3, 4, 21, 22]. In the present study we observed that ucMSC are very susceptible to treatment by IFN-γ and MC, whereby they are induced into a strong immunomodulatory phenotype with high IDO activity, PD-L1 expression and potential to inhibit lymphocyte proliferation. Interestingly, in contrast to IFN-γ treatment, MC treatment also decreases the susceptibility of ucMSC to NK cell lysis and ameliorates inflammation in liver tissue slices.

Our results demonstrate that the phenotype and function of ucMSC is not fixed. Recently, we observed that the lung microvasculature wherein MSC get trapped initiate upregulation of various pathways in MSC and thus alter their phenotype after infusion [42]. It is conceivable that the induced changes by the various pre-treatments will be influenced after infusion. The interactions between pre-treated ucMSC and the microenvironment are therefore a determinant on the effectiveness of these ucMSC.

Efficacy of MSC therapy depends on various aspects, one of which is the biodistribution of MSC after infusion. Although no direct correlation has been established, it is suggested that short-term effects of MSC are mediated by their secretome and their long-term effects are a result of activation and interaction with other cells types, via a hit-and-run mechanism [3, 15, 18, 21, 43]. Thus, the biodistribution of MSC determines which cell types they may encounter and depending on the aim of the therapy a specific distribution will be favoured. Tracking of labelled ucMSC by CryoViz enables in-depth visualization of MSC after administration and subsequently gives an interpretation of not only their biodistribution, but also their survival. Previously, localization in the lungs and short survival time of intravenously injected bone marrow-derived MSC was observed [29]. The present study confirms that the majority of infused ucMSC accumulate in the lungs and that there is a rapid clearance of cells. The retainment of ucMSC was improved by pre-treatment with TGFβ, which corresponds with the observed reduced recognition of ucMSC(TGFβ) by NK cells in vitro. Interestingly, ucMSC(TGFβ) showed no differences in their expression of HLA type I and II whereas they were less recognized less by NK cells. This suggests that TGFβ potentially modulates the expression of ligands on MSC that were not analysed in this study (e.g. NKG2D, DNAM1 and natural cytotoxicity receptors [44–46]). Depending on the therapeutic requirements, longevity or rapid clearance of administered MSC may be desirable. Our results demonstrate that longevity and biodistribution of MSC can be modulated by pre-treatment of the cells.

Lack of immunomodulation by any of the pre-treated ucMSC in vivo could be credited to the fact that the ucMSC were unable to travel towards the liver. As for when the cells were in contact with targeted liver tissue, in the inflammatory ex vivo model, strong amelioration of the inflammation by ucMSC(MC) was observed. Possibly, this in vivo inflammatory liver model requires ucMSC to be in direct contact with the tissue for them to be able to exert their immunomodulatory capacities. Therefore for future studies, administration of ucMSC via the portal vein or hepatic artery may be a more efficient approach for treatment of liver inflammation.

## Conclusions

To conclude, these findings demonstrate that ucMSC are responsive to in vitro treatment, whereby their immunomodulatory properties and their immunogenicity can be differentially modulated. TGFβ treatment of ucMSC reduced their immunogenicity and altered their biodistribution and IFN-γ treatment enhanced their immunomodulatory capacities. Most interestingly, treatment with the multiple cytokine combination MC resulted in reduced immunogenicity, increased immunomodulatory capacity as well as enhanced effectiveness to ameliorate liver inflammation. Therefore, in vitro pre-treatment of ucMSC MC will make them more suitable as an effective immunotherapy targeted for liver inflammation.

## Additional files

Additional file 1: Figure S1. Study design. This study is organized in three sections: in vitro, in vivo and ex vivo. (TIF 6664 kb)

Additional file 2: Table S1. Key immunogenic and immunomodulatory molecules after 3 days of in vitro treatment of ucMSC. The table displays the mean ± SEM percentages of MSC expressing surface markers HLA class –I and –II, PD-L1 and CD73, measured via flow cytometric analysis. Mean ± SEM concentration of L-Kynurenine [µM] and PGE2 [ng/ml]. Fold increase of IL1RA gene expression compared to unstimulated ucMSC. n=5, no significant differences were observed. (DOCX 17 kb)

Additional file 3: Figure S2. In vivo experimental schemes. (*Top*) In the first set of experiments mice were treated with CCl_4_ and 3 hours later pre-treated ucMSC (untreated, IFN-γ, IFN-β, TGFβ, starvation, vitamin B_6_, Starv + VitB_6_, RA and MC), which were labeled with Qtracker605 beads, were infused IV. Four hours after ucMSC infusion the mice were sacrificed and prepared for imaging. (*Bottom*) In the second set of experiments mice were sacrificed 72 hours after CCL4 injection and serum was collected. (TIF 7779 kb)

Additional file 4: Figure S3. Ex vivo experimental scheme. UcMSC were pre-treated for 3 days. Medium was then refreshed and the cells were left overnight in the incubator. The following day, livers were collected from healthy C57BL/6 mice and directly after isolation cut into slices (diameter = 1 cm and thickness = 150 μm) and placed in the wells on top of the pre-treated ucMSC in the presence of LPS. The liver slices were left overnight in the incubator on a shaker (50 rpm). After 24 hours, supernatant and liver slices were harvested. (TIF 4600 kb)

Additional file 5: Figure S4. Characterization ucMSC by flow cytometry. Representative histograms of expression of MSC markers CD13, CD73, CD90, CD105 and negative expression of the endothelial marker CD31 and hematopoietic marker CD45. Stained ucMSC (*grey*) and isotype control (*white*). (TIF 30000 kb)

Additional file 6: Figure S5. MSC diameter. Measured diameter of pre-treated ucMSC in micrometres. Results are shown as means ± SEM (*n* = 5). ^*^Indicates *p* < 0.05. (TIF 4687 kb)

## Abbreviations

ALT: Alanine transaminase
bFGF: Basic fibroblast growth factor
CCl_4_: Carbon tetrachloride
CXCL1: Chemokine (C-X-C motif) ligand 1
FBS: Fetal bovine serum
HLA: Human leukocyte antigen
IDO: Indoleamine 2,3 dioxygenase
IFN-γ: Interferon gamma
IL: Interleukin
IP: Intraperitoneal
IP-10: Interferon gamma-induced protein 10
IV: Intravenous
LPS: Lipopolysaccharide
MC: Multiple cytokine cocktail
MCP-1: Monocyte chemotactic protein-1
MFI: Median fluorescence intensity
MSC: Mesenchymal stromal cells
NK: Natural killer cells
PBMC: Peripheral blood mononuclear cells
PBS: Phosphate-buffered saline
PD-L1: Programmed cell death ligand 1
PGE_2_: Prostaglandin E_2_
RA: Retinoic acid
Starv: Starvation, serum deprivation
TGFβ: Transforming growth factor beta
TNF-α: Tumour necrosis factor alpha
ucMSC: Umbilical cord-derived mesenchymal stromal cells
VitB_6_: Vitamin B_6_, pyridoxal hydrochloride
VitD_3_: Vitamin D_3_, 1α,25-Dihydroxyvitamin
