# Supplementary figures and images for: Cytokine treatment optimises the immunotherapeutic effects of umbilical cord-derived MSC for treatment of inflammatory liver disease
Source: Stem Cell Res Ther. 2017 Jun 8;8:140. doi: 10.1186/s13287-017-0590-6 (PMC5465593; doi:10.1186/s13287-017-0590-6)

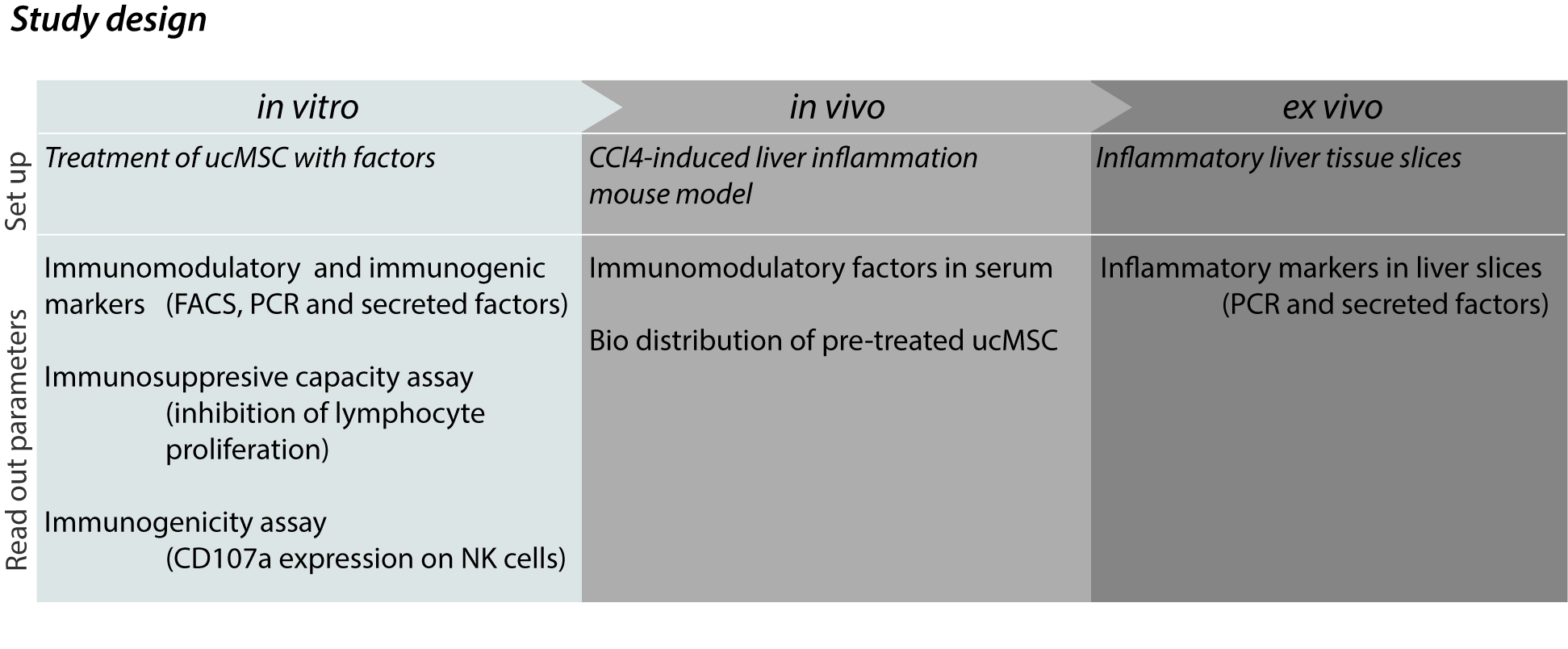

Supplement: Supplementary file 1 — Study design. This study is organized in three sections: in vitro, in vivo and ex vivo. (TIF 6664 kb) [file 13287_2017_590_MOESM1_ESM.tif]

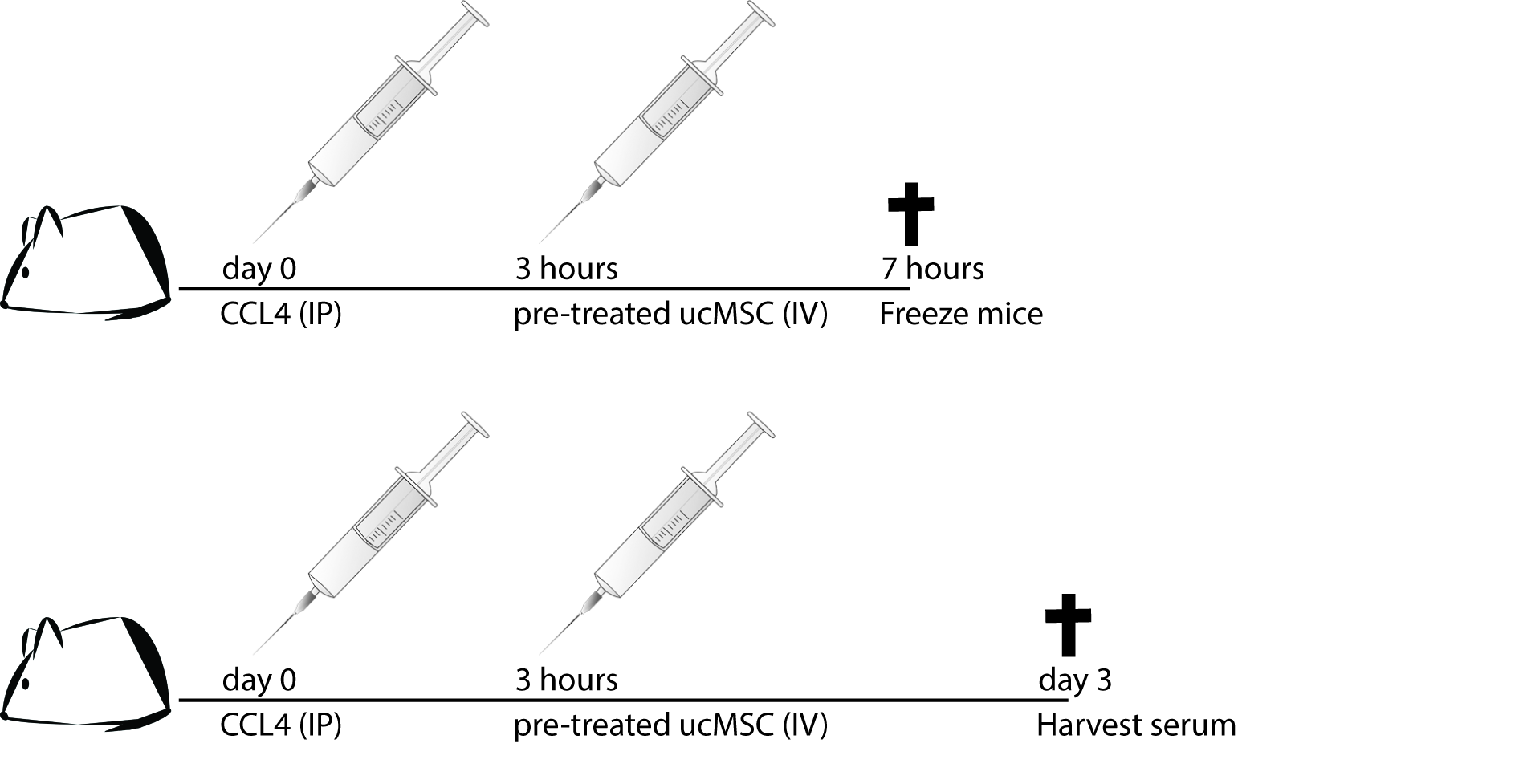

Supplement: Supplementary file 3 — In vivo experimental schemes. (Top) In the first set of experiments mice were treated with CCl4 and 3 hours later pre-treated ucMSC (untreated, IFN-γ, IFN-β, TGFβ, starvation, vitamin B6, Starv + VitB6, RA and MC), which were labeled with Qtracker605 beads, were infused IV. Four hours after ucMSC infusion the mice were sacrificed and prepared for imaging. (Bottom) In the second set of experiments mice were sacrificed 72 hours after CCL4 injection and serum was collected. (TIF 7779 kb) [file 13287_2017_590_MOESM3_ESM.tif]

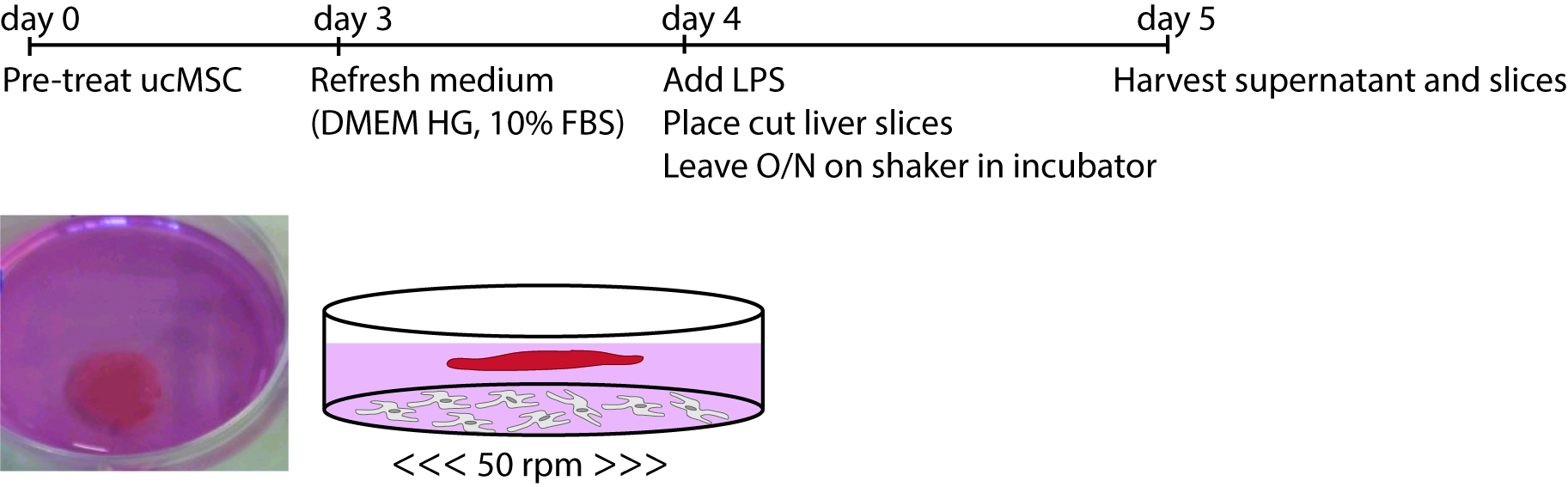

Supplement: Supplementary file 4 — Ex vivo experimental scheme. UcMSC were pre-treated for 3 days. Medium was then refreshed and the cells were left overnight in the incubator. The following day, livers were collected from healthy C57BL/6 mice and directly after isolation cut into slices (diameter = 1 cm and thickness = 150 μm) and placed in the wells on top of the pre-treated ucMSC in the presence of LPS. The liver slices were left overnight in the incubator on a shaker (50 rpm). After 24 hours, supernatant and liver slices were harvested. (TIF 4600 kb) [file 13287_2017_590_MOESM4_ESM.tif]

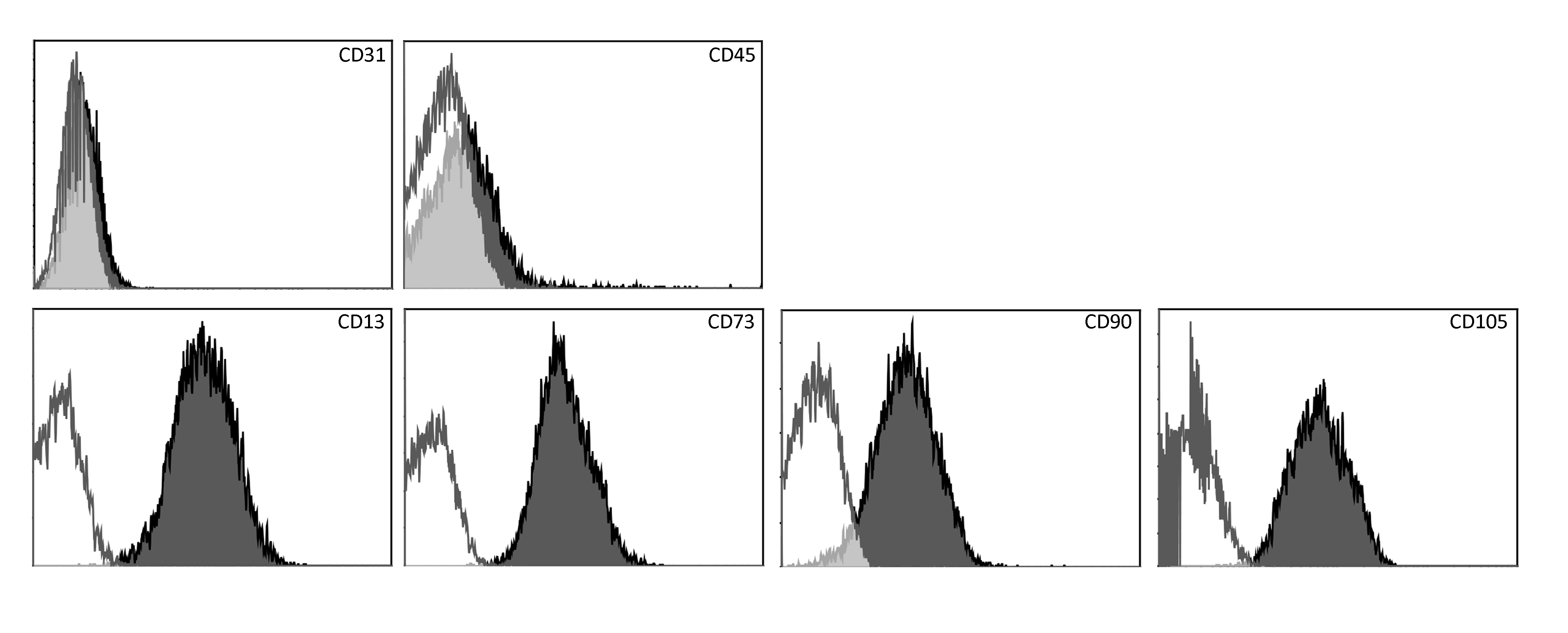

Supplement: Supplementary file 5 — Characterization ucMSC by flow cytometry. Representative histograms of expression of MSC markers CD13, CD73, CD90, CD105 and negative expression of the endothelial marker CD31 and hematopoietic marker CD45. Stained ucMSC (grey) and isotype control (white). (TIF 30000 kb) [file 13287_2017_590_MOESM5_ESM.tif]

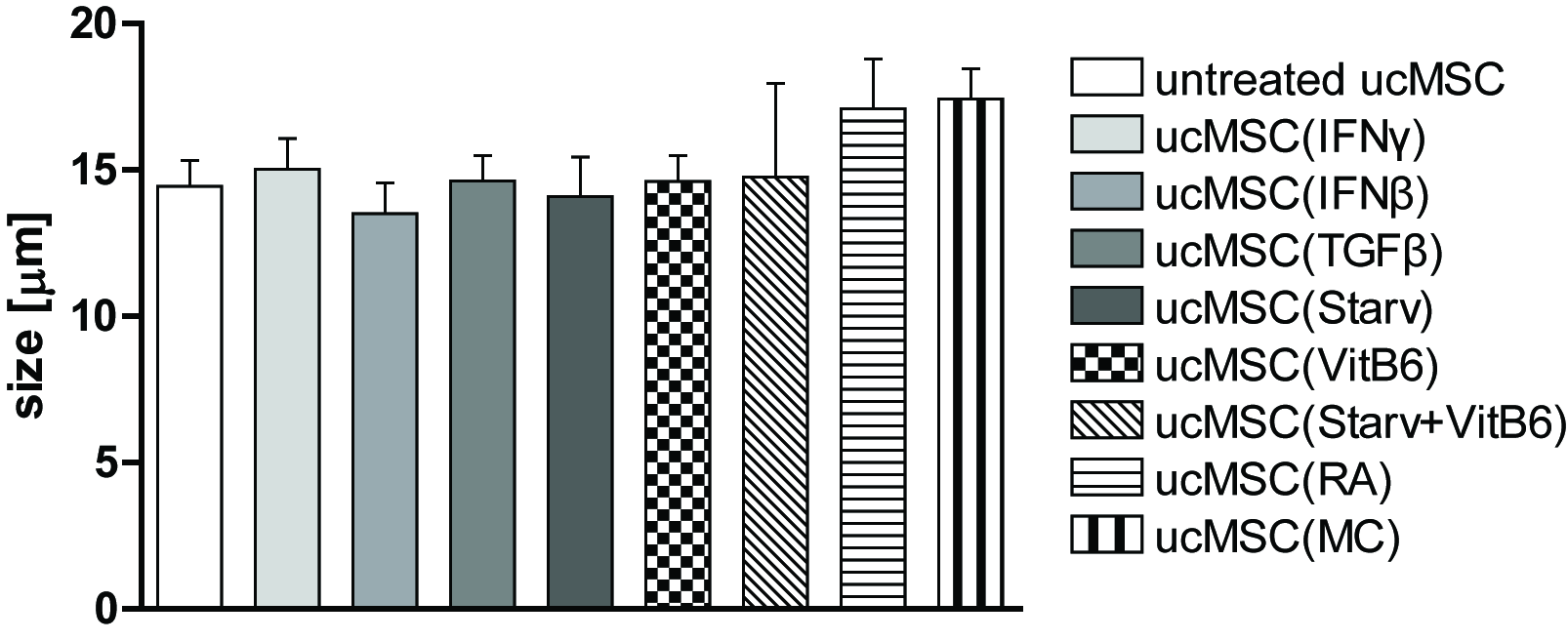

Supplement: Supplementary file 6 — MSC diameter. Measured diameter of pre-treated ucMSC in micrometres. Results are shown as means ± SEM (n = 5). *Indicates p < 0.05. (TIF 4687 kb) [file 13287_2017_590_MOESM6_ESM.tif]
