# Supplementary material for: Cytokine treatment optimises the immunotherapeutic effects of umbilical cord-derived MSC for treatment of inflammatory liver disease
Source: Stem Cell Res Ther. 2017 Jun 8;8:140. doi: 10.1186/s13287-017-0590-6 (PMC5465593; doi:10.1186/s13287-017-0590-6)
Supplement: Supplementary file 2 — Key immunogenic and immunomodulatory molecules after 3 days of in vitro treatment of ucMSC. The table displays the mean ± SEM percentages of MSC expressing surface markers HLA class –I and –II, PD-L1 and CD73, measured via flow cytometric analysis. Mean ± SEM concentration of L-Kynurenine [µM] and PGE2 [ng/ml]. Fold increase of IL1RA gene expression compared to unstimulated ucMSC. n=5, no significant differences were observed. (DOCX 17 kb) [file 13287_2017_590_MOESM2_ESM.docx]

***Table S1 Key immunogenic and immunomodulatory molecules after 3 day in vitro treatment of ucMSC.***

|  | **Immunogenic read out parameters** | | **Immunomodulatory read out parameters** | | | | |
| --- | --- | --- | --- | --- | --- | --- | --- |
|  |  |  |  |  |  |  | *Fold Increase of Gene expression compared to unstimulated* |
|  | **HLA class I expressi-ng cells [%]** | **HLA class II expressi-ng cells [%]** | **L-Kynurenine (correlated IDO activity) [µM]** | **PGE_2_ [ng/ml]** | **PD-L1 expressi-ng cells [%]** | **CD73 expressi-ng cells [%]** | **IL-1RA [corrected for GAPDH]** |
| **[-]** | 7±1 | 10±3 | 2±5 | 14±3 | 46±6 | 95±2 | 1±0 |
| **Vitamin D3** | 13±5 | 15±6 | 2±1 | - | 19±6 | 100±0 | NAV |
| **IL7** | 6±2 | 4±0 | 2±4 | - | 38±8 | 80±10 | NAV |
| **IL15** | 3±1 | 4±0 | -1±0 | - | 43±11 | 76±14 | 1.1±0 |
| **IL17** | 3±1 | 4±1 | 2±1 | - | 41±8 | 74±13 | 1.0±1 |
| **Budesonide** | 10±2 | 20±5 | 2±2 | - | 18±3 | 99±0 | NAV |
| **Trespostinil** | 15±6 | 17±6 | 3±0 | - | 21±6 | 100±0 | 1.1±0 |
| **Activin A** | 14±5 | 14±5 | 2±1 | - | 19±5 | 99±0 | 1.7±1 |
| **TNFα** | 11±4 | 13±5 | 3±4 | - | 21±1 | 99±0 | 1.1±0 |

The table displays the mean ± SEM percentages of MSC expressing surface markers HLA class –I and –II, PD-L1 and CD73, measured via flow cytometric analysis. Mean ± SEM concentration of L-Kynurenine [µM] and PGE_2_ [ng/ml]. Fold increase of IL1RA gene expression compared to unstimulated ucMSC. n=5, no significant differences were observed.
